# An innovative and comprehensive approach to community health and research engagement using the Social Ecological Model

**DOI:** 10.1017/cts.2026.10795

**Published:** 2026-07-17

**Authors:** Farra Kahalnik, Saundra Pennington, Libby Gracia, Emilie Ruiz, Christelle King, Robin T. Higashi, Heather Kitzman, Meera J. Patel

**Affiliations:** 1 Peter O’Donnell Jr. School of Public Health, UT Southwestern Medical Center, Dallas, TX, USA; 2 Harold C. Simmons Comprehensive Cancer Center, UT Southwestern Medical Center, Dallas, TX, USA

**Keywords:** Community engagement, community-engaged research, health equity, diversity, translational science

## Abstract

Equitable participation in clinical research is essential to generating broadly generalizable findings and advancing public health. Yet persistent sociodemographic disparities, driven by mistrust, limited access, and inadequate community engagement, continue to exclude underrepresented racial and ethnic groups from research. To address these barriers, the University of Texas Southwestern Medical Center developed a comprehensive, multi-component community engagement model designed to build trust and enhance research readiness among UREG. The model integrates eight synergistic programs aligned across the five-level Social Ecological Model, including HealthStreet screening events, a Health Needs Assessment, the Community Research Registry, the North Texas Community Health Coalition, Community Engagement Grand Rounds, the Spanish Language Resource, the Community Advisory Panel, and a Community Health Grant Program. These initiatives collectively provide culturally informed outreach and structured pathways for bidirectional input from community members. Since 2023, the model has achieved substantial impact, including 5069 community-based health screenings, over 100 consultations to investigators, and more than $44 million in funded grants. Findings demonstrate that sustained, multi-level engagement can strengthen trust, expand research access, and support more equitable translational science.

## Introduction

Participation in clinical research is considered a social good because it leads to biomedical discoveries that prevent and treat disease, ultimately improving public health. Even when clinical research does not directly impact individual research participants, it is described in the research ethics literature as having social value [1]. Anyone who has received evidence-based healthcare is the beneficiary of the knowledge gained through clinical research, and only by promoting a culture that recognizes its importance within healthcare delivery systems will it achieve its full potential [2]. Despite its social value, only about 5% of American adults have ever participated in a clinical trial and significant sociodemographic disparities exist, with participants more likely to be older, female, non-Hispanic White, and more educated [3].

When patient diversity in clinical trials is limited, results may not be generalizable, hindering equitable and effective research translation to the broader community. Specifically, research participation among underrepresented racial and ethnic groups (UREG) is significantly lower than non-minority populations; for example, African American patients were underrepresented in NCI sponsored cancer clinical trials from 2000 to 2002 [4]. Unfortunately, these underrepresented individuals are typically part of high-risk communities that face the greatest health challenges but benefit the least from research discoveries, further exacerbating existing health disparities. Clark et al. identified five barriers to research participation among UREG, including mistrust, lack of comfort with the clinical trial process, lack of information about clinical trials, time and resource constraints associated with participation, and lack of clinical trial awareness [5]. A qualitative study that assessed barriers and facilitators for reaching UREG populations in clinical research found that mistrust was an overarching theme across the Social Ecological Model including individual, interpersonal, community and systemic level factors [6, 7].

Mistrust of clinical research, especially among the African American community, stems from a long-standing history of institutionalized racism in American medicine and oftentimes continues and is further worsened by ongoing discrimination [8]. However, barriers associated with a lack of information or awareness must also be considered. Remarkably, many individuals have never been approached to participate in research or have been recruited through culturally or linguistically insensitive approaches that inhibit fully informed decision-making. Only 9% of all Americans have ever been invited to participate in a clinical trial, of which less than half went on to participate [9]. Even within this small population, certain demographic and clinical characteristics – being a non-Hispanic Black person, having a college education, being single, urban-dwelling, or having at least one medical condition – increased the likelihood of clinical trial *invitation*; however, non-Hispanic Black persons had lower odds of *enrolling* in clinical trials as compared to non-Hispanic Whites [9].

Initiatives to improve diversity in research should be conceived and implemented through the practice of sustained community engagement. This is the process of working collaboratively with groups of people affiliated by geographic proximity, special interests, or similar life experiences with respect to issues affecting their well-being [10]. Strategies that engage with UREG are needed to build trust in clinical research and medical institutions as well as improve access to research through inclusive and equitable outreach practices [11]. Clinical trial site readiness practices that promote diversity, equity, inclusion, and access include engaging communities early and incorporating community members to serve as advisors; hiring and supporting diverse staff; making efforts to improve recruitment, retention, and follow-up for underrepresented communities; employing clear and culturally appropriate privacy and security protections for participants; and ensuring data collection and analysis practices are unbiased [12]. Such multidimensional strategies may address and disentangle the complex interplay of barriers that contribute to the enduring challenges of underrepresented communities, bolstering the capacity of research translation to improve public health.

Efforts to improve diversity in biomedical research have justifiably become a priority across academia and industry. However, when such efforts are ineffectively executed, or top-down initiatives fail to build sustainable relationships, mistrust among these communities deepens [13]. Community-focused, trust-building approaches driven by community priorities are needed to resolve this challenge. The Clinical and Translational Science Award (CTSA) program, launched by the NIH in 2006, provides an infrastructure within academic health centers to support and scale community-engaged research practices [14]. Since then, a wide array of community engagement activities across the CTSA national consortium have been implemented. However, despite the abundance of published literature showing that community engagement improves the integration of UREG populations into clinical trials, studies depicting the effectiveness of different community engagement strategies or a comprehensive model of sustainable community engagement remains limited [15].

## Methods

### The UT Southwestern Medical Center (UTSW) CTSA Program Community Engagement Model

The UT Southwestern Medical Center (UTSW) in Dallas, Texas has developed and implemented a comprehensive multi-component community engagement model to bolster the reach and efficiency of translational research. The Office of Community Health and Research Engagement (OCHRE) operates an infrastructure comprised of eight integrated programs, achieving impact across the five-level Social Ecological Model to address the interplay between individual, community, and the physical, social, and political environments in which one exists [16] (see Table 1).

**Table 1.** Alignment of community engagement strategies across levels of the Social Ecological Model Table 1 long description.

	**Intrapersonal** -Knowledge -Attitudes -Perceptions	**Interpersonal** -Family -Peers -Social networks	**Organizational** -Churches -Community organizations -Academic health centers	**Community** -Neighborhoods -Relationships among organizations -Social/cultural norms	**Policy** -Local -State -Federal
**HealthStreet screening events**	Increases individual knowledge and builds trust through outreach events, health screenings, and health education.	Enhances influence via social networks through holding events in community settings where families/social groups convene.	Builds capacity among local organizations through conducting health screenings for their constituents.	Improves community trust in our academic health center by offering free services and being a consistent presence.	
**Health Needs Assessment (HNA) **	Increases perceptions that individual community members’ needs are valued and heard by asking questions about health behaviors and attitudes.		Gathers data that has the potential to inform health interventions among community organizations.	Provides data to understand community level needs and concerns.	Gathers data that has the potential to inform health policies at the local level.
**Community Research Registry (CRR)**	Increases individual knowledge and builds trust by providing research participation opportunities and education.		Builds capacity at the academic health center level to implement trials with diverse representation, leading to more generalizable results and equitable treatment/interventions.	Provides information to members on local activities, events, and webinars.	Improves research translation to the broader community through policy changes at all levels.
**North Texas Community Health Coalition (NTCHC)**			Increases organizational capacity through resources, education, and networking opportunities.	Improves organizations’ capacity to provide health services and resources more efficiently by breaking down silos and encouraging collaboration.	Provides an infrastructure well suited to advocacy for local policy change.
**Community Engagement Grand Rounds**	Increases knowledge about health issues impacting disadvantaged communities, best practices for community-engaged research – targeting both researchers and community stakeholders.			Emphasizes the importance, among the research community, of incorporating community engagement practices into research at all stages.	
**Spanish Language Resource (SLR)**	Increases investigators’ awareness of the scientific and ethical value of inclusion of Spanish speakers in research using accurate and culturally appropriate study materials.	Facilitates enhanced communication and engagement among investigators, potential study participants, and their families about participation in research.	Reduces translation cost barriers for early-stage investigators to conduct research with Spanish-speaking populations.	Shows respect for Spanish-speaking community members by ensuring that study materials are equitable.	Adheres to the principles of justice and beneficence toward human subjects in research as outlined in the Belmont Report.
**Community Advisory Panel (CAP)**	Increases research-related literacy and empowerment.	Provides a social/support network for members engaged in providing feedback on research.	Assists investigators in integrating the community perspective into their research.	Ensures that research practices reflect the needs, preferences and cultural norms of the community.	
**Community Health Grant Program**			Supports and builds capacity for meaningful community–academic partnerships between community organizations and academic researchers.	Demonstrates a commitment on the part of academic health centers to support these types of partnerships, leading to changing norms/perceptions at the community level.	Produces evidence that can inform policy change.

### HealthStreet

The HealthStreet program strives to reduce disparities in healthcare and research by linking community members to social and medical services and directly connecting them to research opportunities. In collaboration with HealthStreet’s founder [17], UTSW launched the outreach program in 2023 tailoring it to previous work conducted by OCHRE [18–20] and UTSW [21].

HealthStreet utilizes a team of Community Health Workers (CHWs), whose training and trusted community relationships make them ideally suited to conduct community outreach. A proof-of-concept study conducted at six CTSA institutions across the USA found the model of combined social service and research navigation by CHWs to be effective in building trust in the community, thereby motivating people to participate in health research [22].

As a consistent presence and source of support in the community, HealthStreet builds and sustains the trust needed to engage those typically underrepresented in research. HealthStreet works synergistically with the North Texas Community Health Coalition (NTCHC) organizations (discussed below) to reach local community members. HealthStreet events are held in community settings (e.g., churches, libraries, barber shops, recreation centers, apartment complexes, health fairs) in partnership with trusted community organization partners. Reaching individuals where they live, work, worship and play addresses social determinants of health (SDoH) barriers such as transportation, access, and time. Furthermore, many studies leverage HealthStreet services provided in community settings to recruit and conduct initial screenings to determine research eligibility [23].

#### Free Point-of-Care Health Screenings

During the HealthStreet interaction, community members are offered one or more free, point-of-care health screenings. These may include blood pressure (Omron 3 Series Upper Arm), diabetes (HbA1c, Siemans DCA Vantage HbA1c Analyzer), chronic kidney disease (urine albumin-to-creatinine ratio [uACR] Clarity CLA-U120C), and cardiovascular disease (LDL, HDL, total cholesterol, Cholestech LDX Professional Lipid Testing). Those with elevated screening results are provided with clinic/social service referrals and health education materials.

#### Health Needs Assessment (HNA)

Individuals are invited to complete a HealthStreet Health Needs Assessment (HNA) survey, which collects demographics, health concerns, lifestyle behaviors, employment, food insecurity, housing, insurance status, spoken languages, mental health, and perceptions of clinical research, empowering community members to share their perspectives to guide health research and policies. The HNA is also used by CHWs to provide personalized referrals for healthcare and SDoH needs (e.g., food access, benefit enrollment, housing, mental health clinics, primary care).

#### Community Research Registry (CRR)

As part of the HNA, individuals can opt to join the Community Research Registry (CRR). This opens a conversation about the importance of diverse representation in clinical research and provides a mechanism to disseminate research opportunities, public health, and community programming via a monthly newsletter.

**Figure 1.**
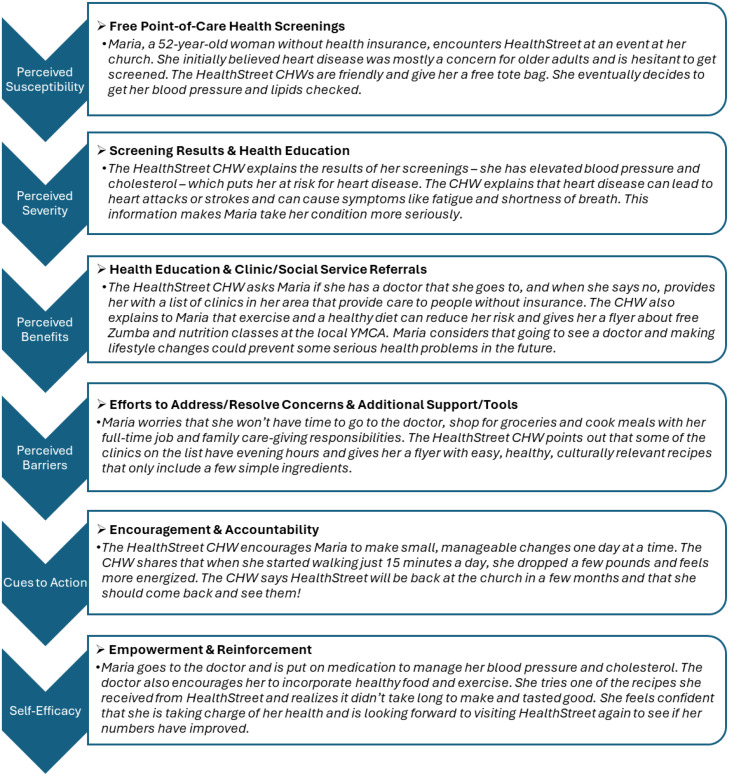
Figure 1 long description. Example of the Health Belief Model used to guide a HealthStreet encounter.

The engagement strategies of HealthStreet are theoretically guided by the Transtheoretical Model of Change (TTM) and Health Belief Model (HBM) [24]. By utilizing the TTM, CHWs approach people based on their current stage of readiness to change – precontemplation, contemplation, preparation, action, or maintenance – guiding CHW prompts to meet individuals where they are. HBM variables, such as susceptibility and severity, are addressed with point-of-care testing and health education, whereas the benefits of avoiding the threat and factors influencing the decision to act (barriers, cues to action, and self-efficacy) are integrated into CHW navigation and counseling. Figure 1 illustrates the HBM strategies HealthStreet CHWs utilize to promote healthy behaviors.

### North Texas Community Health Coalition (NTCHC)

**Figure 2.**
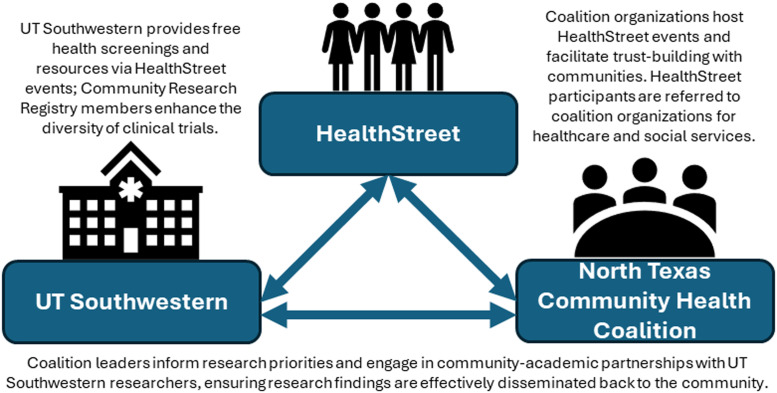
Figure 2 long description. Relationship between North Texas Community Health Coalition, HealthStreet, and UT Southwestern.

The NTCHC bridges multisector community leaders and stakeholders committed to improving the health of North Texas communities. It provides a forum to discuss community health challenges and inform UTSW’s clinical and translational research priorities, setting a foundational infrastructure through which HealthStreet can reach UREG and medically underserved communities. The NTCHC is supported by the Community Coalition Action Theory (CCAT), which posits that a community coalition is a structured, formal, multipurpose, and long-term alliance, typically local or regional in scope, in which all members work together toward a common purpose [25]. According to the CCAT, community coalition development is dynamic; coalitions progress through stages from formation to institutionalization, with frequent loops back to earlier stages as new members join and new issues arise. The NTCHC was purposely designed to meet the needs of community partners including: (1) grant writing support, (2) quarterly luncheons with guest speakers and networking time, (3) an annual symposium based on the NTCHC’s choice of topic, and (4) free HealthStreet screening events for their clients and/or population. Figure 2 illustrates the synergistic relationship between the NTCHC, HealthStreet, and UTSW that facilitates translational science.

### Community Engagement Grand Rounds

The Community Engagement Grand Rounds lecture series provides educational opportunities demonstrating best practices for the integration of community engagement activities into research to build sustained, community–academic partnerships. This series shares current examples, methods, and results of community engagement and provides concrete steps to engage a community across the research life cycle. The series was initially conceived as an educational resource for academic researchers, but over time it has incorporated topics that aligned with the interests of community partners.

### Spanish Language Resource (SLR)

The SLR, a service now provided through the O’Donnell School of Public Health at UTSW, aims to ensure that research materials are accurate and culturally appropriate for Spanish-speaking study participants by providing translation and cognitive response testing services to investigators. This includes measuring and recommending language in accordance with best practice [26] and, given that most US adults read at approximately an 8^th^ grade reading level, at a low literacy level (<9th grade). The SLR also uses “neutral Spanish” that can be understood by Spanish speakers from different countries and cultural backgrounds. SLR services are evidence-based and performed by native speakers and individuals certified in verbal, written, and medical Spanish proficiency.

1. Translation: Basic English to Spanish translation requests undergo a three-step process consisting of human translation, editing, and proofreading (TEP) [27]. The TEP approach balances the need for efficiency with the goal of accuracy through consensus-building [28]. It prioritizes cultural meaning over literal translation and is adapted from the WHO’s translation and linguistic evaluation best practices [29].
2. Cognitive response testing (a.k.a. cognitive interviewing): When more conceptually complex translations are required, SLR uses back-translation and cognitive interviewing, a qualitative research technique, to augment the TEP process. By probing individuals’ understanding of the meaning of concepts and words, cognitive interviews allow investigators to pretest study materials, which can be especially valuable when adapting novel survey items or modifying scientifically validated instruments for lower-literacy populations [30].

The CTSA subsidizes the cost for SLR services for new investigators who lack external funding so that cost does not prevent the inclusion of Spanish speakers in research. Removing the potential cost barrier promotes more equitable representation and enhances the scientific rigor of research by increasing the generalizability of findings.

### Community Advisory Panel (CAP)

Modeled after the Community Engagement Studios of the Meharry Vanderbilt Community Engaged Research Core [31], the CAP was designed to provide investigators with timely feedback from community stakeholders on study feasibility and design, cultural appropriateness, participant recruitment, program implementation and dissemination. The one-hour facilitated feedback sessions involve bidirectional communication and co-learning between researchers and community members. CAP members are typically patients, caregivers, or members of UREG who provide feedback on how current research can be improved to meet community needs. Each CAP member receives a meal and $60 per hour in compensation.

The CAP was initially comprised of a core group of 10 older adult members who were patients at the local safety-net health system, Parkland Health, in 2022. Over the course of approximately two years, two members were lost and 15 gained (for a total of 23 current members). New members were recruited with the intent to increase the demographic diversity of the panel and incorporate caregivers of children who would be willing to advise on pediatric-specific studies. The CAP is remarkably diverse, with a racial/ethnic composition of 48% African American (11), 17% Hispanic (4), 9% Middle Eastern (2), and 26% White (6), and a gender composition of 78% female (18) and 22% male (5).

Based on requests from researchers focused on pediatric populations, the Youth CAP was launched in January 2025 with volunteers (ages 15–18) from Dallas’ local school district’s health professions magnet high school. The Youth CAP includes 10 members: 8 female/2 male; 3 African American, 7 Hispanic.

### Community Health Grant Program

The Community Health Grant Program aims to encourage collaborative identification of community health priorities and foster sustainable community–academic partnerships through two funding mechanisms – the Planning Grant and Pilot Grant – which offer up to $15,000 and $30,000, respectively, in annual funding. The Planning Grant supports the planning and development of new, innovative, and impactful research projects that are conducted in community-based and/or clinical settings and have the potential to lead to future community-engaged research projects. Study teams are required to complete the CITI Program course “Community-Engaged and Community-Based Participatory Research” and are offered training and mentorship throughout the grant year to facilitate development of meaningful research questions, scientifically rigorous research protocols and successful partnerships that align with accepted principles of community engagement. Upon completion of Planning Grant deliverables and obtaining IRB approval (if applicable), Planning Grant awardees are eligible to apply for Pilot Grant funding during the next grant cycle. However, Pilot Grant eligibility is not dependent upon completion of the Planning Grant phase. Over the last four years, a total of $299,796 has been awarded in Planning and Pilot Grants. The extramural funding received as a result of these grants from the first two years was $2,289,105, (a 1172% return on investment), which has a significant impact on building the portfolio of community-engaged research at UTSW and affiliated institutions.

## Results

### HealthStreet

**Figure 3.**
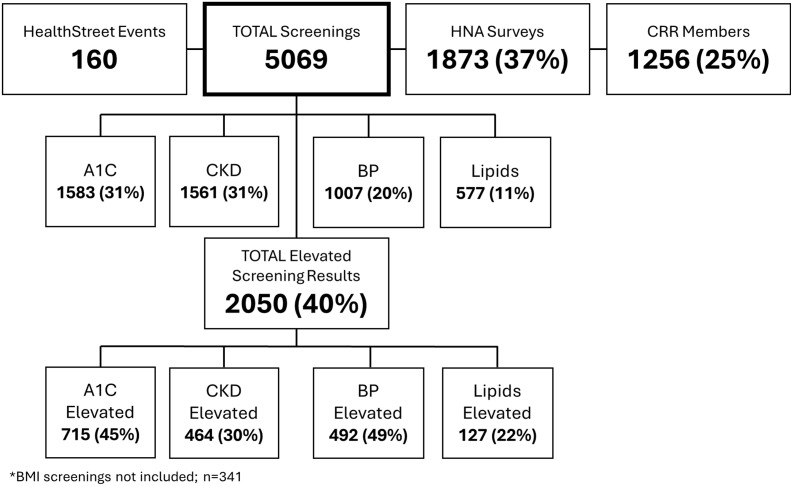
Figure 3 long description. HealthStreet outcomes*.

Since its inception in April 2023, the HealthStreet program has conducted 160 events and 5069 health screenings. See Figure 3 for the total number and percentage of individuals screened who completed the HealthStreet Survey, joined the CRR, and a breakdown of the number and percentage of screenings by type and elevated results. Notably, 25% of individuals encountered through HealthStreet elect to join the CRR, underscoring the effectiveness of our engagement approach. Approximately 10 studies have used HealthStreet to screen and recruit for studies either in person at events or contacting individuals who joined the CRR.

### North Texas Community Health Coalition (NTCHC)

We have held 10 quarterly luncheons and approximately 15 researchers have requested collaborative partnerships with members. Over the past three years, the NTCHC has grown from 10 to 60 organizations primarily through word of mouth. Further, we have held three annual symposiums with a high level of in-person attendance each year (2024: *n* = 144; 2025: *n* = 136; 2026: *n* = 116). At the June 2024 lunch meeting, partner organizations reported benefiting from coalition membership through grant writing support and inclusion in grant opportunities, networking with other community organizations – particularly in-person – and access to UTSW healthcare experts and researchers.

### Community Engagement Grand Rounds

Since the inception of the series in March 2022, 14 lectures have been delivered by a combination of local and national speakers, which included a total of 929 attendees.

### Spanish Language Resource (SLR)

Since late 2022, the SLR translated approximately 400 pages of documents consisting of recruitment scripts, participant information sheets, surveys, interview guides, recipes, and a complete training manual and client workbook. Studies tackled compelling health issues such as identifying preferences among food pantry clients, optimizing outcomes before and after liver transplantation in a safety-net setting, and adapting a psychoeducational training to enhance positive mood and well-being to a Spanish-speaking population.

Study investigators were asked to confidentially complete surveys rating their satisfaction with SLR services upon return of their translated documents. On a 5-point Likert scale, most survey respondents (*n* = 20) were “very satisfied” with the service quality (100%), timeliness (90%), and overall (95%). One survey respondent said:

*We liked the explanation of nuance around phrases and words that don’t directly translate.*

Within six months, investigators who used the SLR produced one manuscript, two posters, one conference presentation, and one White Paper for a community organization partner. All anticipated submitting additional peer-reviewed publications, and three investigators already acquired additional funding based on the success of the study for which they used SLR-translated materials. As one investigator added,

*Translation services are a necessity for my research as over 40% of our study population prefers to communicate in Spanish. I appreciate that this service is offered at UT Southwestern and is affordable to researchers. Continued support through the CTSA to keep costs low makes research feasible for early-stage investigators (& all investigators, really!).*

### Community Advisory Panel (CAP)

To date, 24 (22 adult; 2 youth) CAP sessions have been held with research teams from UTSW and affiliated institutions. According to satisfaction surveys gathered from CAP members following each session, most CAP members “strongly agreed” (based on a 5-point Likert scale, *n* = 114 responses) with each of the seven statements. For example, “The presenter provided enough information for me to understand the topic for discussion” (85% strongly agreed); “I was able to express my thoughts and opinions about the topic” (87% strongly agreed); and “My participation in this meeting felt worthwhile (84% strongly agreed).”

Based on surveys collected from investigators following each session to evaluate their satisfaction with the CAP feedback session, all respondents were “very satisfied” with the overall session and its logistical processes (*n* = 16). When asked, “What aspects of the CAP session were particularly helpful or useful,” responses included:

*It was very helpful to get feedback in the context of each individual’s life experience.*

*The panelists! Concrete recommendations, creative ideas. The organization-arranged around my availability, multiple dates provided, and prompt follow up by the organizer.*

To assess the longer-term impact of the program, 6-and 12-month investigator follow-up surveys are disseminated. When asked “Please share with us how your CAP consult impacted your project,” one respondent said:

*The CAP feedback was helpful in our development of interview guides for our formative structured interviews. The CAP highlighted the potential importance of the envelope/packaging for the letter. Prior to the CAP, we were focused only on the letter itself. As a result, we added questions to our interview guide about the envelope/packaging to gather input on this for development of the interview and outreach strategy.*

Based on the feedback summarized above, the CAP program is a meaningful experience for the CAP members and an invaluable resource for investigators.

### Community Health Grant Program

Feedback surveys are collected from both the research teams and their community partners at the 6-month mark of the program. When researchers were asked (*n* = 9, based on a 5-point Likert scale), “In the last 6 months, how has your overall experience been with your community partner?” 78% responded “Excellent,” 11% responded “Fair,” and 11% did not respond. When asked to elaborate, one respondent said:

*We are fortunate to be building on a longstanding partnership with the organization, and this has enabled honest feedback and insight from the partner about their needs/client needs from the beginning. This allowed us to hit the ground running. We feel that we’ve had an opportunity to meet their needs/desires while also collecting data that informs our equity-based projects.*

When community partners were asked (*n* = 18, based on a 5-point Likert scale), “In the last 6 months, how has your overall experience been with your UT Southwestern research partner/group?” 50% responded “Excellent” and 11% responded “Good,” and 39% did not respond. When asked to elaborate, one respondent said:

*Thought provoking dialog with clinicians on our project. Refreshing to hear the level of interest in our perspective on what would be helpful.*

Based upon the input collected, members of both the research and community sides of these CTSA grant-funded community–academic partnerships have expressed high levels of satisfaction and productivity with the implementation of their collaborative projects.

Overall, the comprehensive community engagement model developed by OCHRE has also supported researchers within UTSW and affiliated institutions through over 100 individualized consults as well as grant applications, contributing to over 50 grant submissions and 16 funded grants totaling $44,759,675. Services included consults, letters of support, CAP feedback for development of research proposals, connection to community partners for grant submissions and implementation, use of HealthStreet for education, outreach, and recruitment, and integration of the NTCHC for partnerships and study implementation. For example, an $18.4 million PCORI grant was awarded after multiple sessions with the CAP, whose invaluable feedback strengthened the design and implementation of a community-based intervention. Further, this model has contributed to the literature of community-engaged research via peer-reviewed papers: 22 published and 7 under review. These accomplishments reflect the program’s growing reach, measurable impact on community health, and its role in advancing both institutional research capacity and community-engaged scholarship.

## Discussion

OCHRE’s community engagement model has seen tremendous success over recent years. A key strength of this model is its integration across multiple social ecological levels, which recognizes that research participation is shaped not only by individual choices, but also by broader social, cultural, and policy environments. The programming appears to create a synergistic effect that promotes both feelings of community trust and institutional capacity for engagement. In the future, we plan to measure community member and organization trust longitudinally, as well as clinical trial accrual resulting from this model, to provide support for best practices in translational science. Despite these advances, limitations remain. Evidence on the relative effectiveness of individual components of this model is limited, and further evaluation is needed to identify which strategies are most impactful in increasing minority participation and sustaining partnerships. Generalizability may also be constrained by institutional resources, requiring adaptations for replication at other CTSA hubs or academic health centers.

Looking ahead, the UTSW model contributes to a growing national conversation about sustainable frameworks for community engagement in research. For researchers and communities, this model demonstrates the value of building trustworthy, long-term partnerships, with a focus on sharing knowledge bidirectionally, rather than transactional interactions. For policymakers and funders, the findings underscore the importance of investing in infrastructure that supports ongoing, sustainable engagement rather than one-off initiatives. Collectively, these insights suggest a pathway where translational research can more effectively advance equity and improve population health.

## Table 1. Long description

The eight community engagement strategies of the Office of Community Health and Research Engagement at UT Southwestern have been aligned with the five levels of the social ecological model. The levels of the social ecological model are presented in row 1 and the community engagement strategies are listed in column 1. The alignment portrayed in this table demonstrates how the activities conducted by the Office of Community Health and Research Engagement bolster the research and efficiency of translational research by impacting the interplay between individual, community, and the physical, social, and political environments in which one exists.

Navigate back to Table 1..

## Figure 1. Long description

The flow diagram illustrates how HealthStreet applies engagement strategies that are theoretically driven by the Health Belief Model. The figure shows an example of a HealthStreet encounter. The Health Belief Model serves as a pivotal foundation for how community health workers approach individuals for navigation and counseling. Depending on where an individual stands, the community health worker may address severity of elevated blood pressure and cholesterol via health education with the screening results or encouragement via sharing ways to make small changes to lifestyle habits.

Navigate back to Figure 1..

## Figure 2. Long description

The image illustrates the triangulation between the North Texas Community Health Coalition, HealthStreet, and UT Southwestern. The synergistic relationship between these three groups facilitates translational science. UT Southwestern provides free screenings and resources via HealthStreet events and HealthStreet enrolls individuals interested in clinical trials into a Community Research Registry. Coalition organizations host HealthStreet events and provide healthcare and social services for referrals. Coalition leaders also inform research priorities and engage in UT Southwestern research, ensuring effective dissemination of research findings to the community.

Navigate back to Figure 2..

## Figure 3. Long description

Out of 160 events, 5,069 total screenings were completed, 1,873 (37%) completed health needs assessments, and 1,256 (25%) enrolled in the community research registry. Of the total screenings, 2,050 (40%) individuals reported elevated results for hemoglobin A1c, risk for CKD, blood pressure, or lipids.

Navigate back to Figure 3..
